# Extreme Physiology Extreme Tolerance to Hypoxia, Hypercapnia, and Pain in the Naked Mole-Rat

**DOI:** 10.1007/s10974-022-09623-3

**Published:** 2022-07-19

**Authors:** Thomas J. Park, Jane Reznick

**Affiliations:** 1 Department of Biological Sciences and Laboratory of Integrative Neuroscience, University of Illinois at Chicago, Chicago, IL United States of America; 2 Cologne Excellence Cluster for Cellular Stress Responses in Aging-Associated Diseases (CECAD), Faculty of Medicine and University Hospital, University of Cologne, Cologne, Germany

**Keywords:** Systems Neurobiology, Hypoxia, Anoxia, Metabolism, Pain

## Abstract

Challenging environmental conditions can drive the evolution of extreme physiological traits. The naked mole-rat has evolved to survive and thrive in a low oxygen, high carbon dioxide environment that would be deadly to humans and most other mammals. The naked mole-rat’s lifestyle is unusual in that this species combines subterranean living and living in large, social groups of up to 300 + individuals. Many respiring animals in a closed environment can lead to depletion of oxygen (hypoxia) and accumulation of carbon dioxide (hypercapnia). Naked mole-rats display a variety of physiological traits that negate the adverse effects of living in this atmosphere. For hypoxia tolerance, naked mole-rats have a low resting metabolism, high affinity hemoglobin, intrinsic brain tolerance, the ability to use fructose for anaerobic glycolysis, and the ability to enter a low energy, suspended animation-like state. For hypercapnia tolerance, these animals have a mutation in a voltage gated sodium channel that effectively eliminates neuronal responses to tissue acidosis. In other mammals, acidosis from exposure to high concentrations of carbon dioxide induces pain and pulmonary edema. Understanding these mechanisms of extreme physiology is not only inherently interesting, but it may lead to biomedical breakthroughs in research on heart attacks, strokes, and pain pathologies.

**Supplementary Information:**

The online version contains supplementary material available at 10.1007/s10974-022-09623-3.

## Introduction

**Fig. 1:**
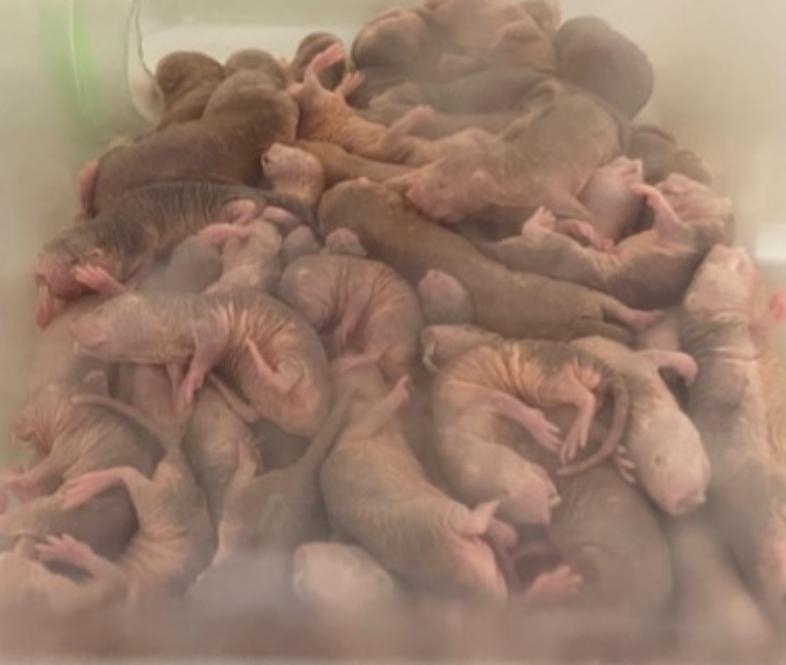
Naked mole-rats spend a great deal of time piled together in communal nest chambers (Zions et al. 2020). This photograph is from a colony at the University of Illinois at Chicago

Naked mole-rats are subterranean rodents native to parts of East Africa. They initially garnered attention because of their extreme sociality (Jarvis 1981). They live in underground colonies that can include up to 300 members. In the laboratory setting, they are well known to gather together in large numbers, literally piling on top of each other (Fig. 1). Within an individual colony, only 1 female and 1–3 males are responsible for breeding. The other adults fall into one of two castes, which are often referred to as a soldier caste and a worker caste, each displaying a different set of behavioral activities (Holmes and Goldman 2021). It should be noted that there is some controversy over the division of labor between these casts (see Gilbert et al. 2020 for a review). The social structure of naked mole-rats is often referred to as eusocial because it resembles the social structure of social insects. Since the initial publication about naked mole-rats living in a eusocial structure (Jarvis 1981), they have become a valuable model animal for scientists studying many aspects of social behavior (Holmes and Goldman 2021).

In addition to extreme sociality, naked mole-rats display a number of other fascination traits. They are extremely long lived (Ruby et al. 2018). In many ways they are heterothermic, taking on the temperature of their environment (Buffenstein et al. 2021 ; Buffenstein and Craft, 2021). They are extraordinarily resistant to cancer (Delaney et al. 2021). They are insensitive to certain types of pain, like pain from acid and capsaicin (Lewin et al. 2021). Finally, they are extremely tolerant to oxygen deprivation (Park et al. 2021).

The last two traits – insensitivity to acid pain and tolerance to oxygen deprivation – will be the focus of the current review article. Both traits are almost certainly related to the atmosphere in which this species has evolved. Living in large colonies is a good strategy for successfully finding widely dispersed tubers (something like a very large potato). In their East African subterranean environment, a small colony would be more challenged by burrowing through the soil to find food. However, large colonies can divide up into multiple digging teams, and then share the food when one team finds a tuber (food-aridity hypothesis; Bennett and Faulkes, 2000). However, there is a substantial negative side effect of many respiring mammals living together in an unventilated burrow depleted of oxygen (O_2_) and high in accumulated carbon dioxide (CO_2_) (Bennett and Faulkes, 2000). Depletion of O_2_, also known as hypoxia, is a challenge for generating enough energy to supply all the cells in the body. Accumulation of CO_2_, also known as hypercapnia, is a challenge because the related tissue acidosis is painful to the eyes and upper respiratory tract, and it is a trigger for pulmonary edema and asphyxiation. Naked mole-rats have a number of extreme physiological traits that allow them to deal with these harsh conditions.

It is important to note that Holtze et al. (2017) found that O_2_ and CO_2_ concentrations in naked mole-rat tunnels were very similar to those of the surface atmosphere. Those authors also suggested that hypoxic and hypercapnic conditions may well be a challenge in communal nest chambers where up to hundreds of individuals huddle together. Zions et al. (2020) showed that captive naked mole-rats spend more than 70% of there time in the nest chamber, which is substantial.

## Extreme Hypoxia Tolerance

### Low temperature, low metabolic rate, and high affinity hemoglobin

Naked mole-rats employ at least two mechanisms to conserve oxygen-dependent energy. The first mechanism involves a reduced generation of body heat when they are within their thermoneutral zone (reviewed in Buffenstein et al. 2021; Buffenstein and Craft, 2021). Within their equatorial burrows, naked mole rats can find a desirable ambient temperature by moving to different depths of the burrow as the surface of the earth heats and cools over the course of the day and night (Jarvis 1990). In other words, they can behaviorally thermoregulate. Holtze et al. (2017) found that burrow temperatures range over 24.6–48.8 °C.

Other mammals use physiological thermoregulation and expend a huge amount of energy to maintain their desired body temperature (Cannon and Nedergaard 2011). Hence, by favoring behavioral thermoregulation, naked mole-rats greatly reduce their need for oxygen-dependent energy (Buffenstein and Yahav 1991). Interestingly, in the laboratory, naked mole-rats exposed to cool temperatures (e.g. 20 degrees C) attempt to physiologically thermoregulate. However, they are unable to maintain their preferred temperature (Oiwa et al. 2020). Also interesting, within captive colonies maintained within their thermoneutral zone, naked mole-rats regulate their body temperatures within 1 degree C by huddling together (Yahav and Buffenstein, 1991). Another way that naked mole-rats behaviorally respond to hypoxia is that they progressively reduce activity in response to progressively more severe hypoxic atmospheres, presumably to save energy (Ilacqua et al. 2017).

Their second energy saver involves the naked mole-rat’s reduced resting metabolic rate, about 2/3rds that of similar-sized rodents (Buffenstein and Yahav 1991). In addition to these oxygen conserving mechanisms, naked mole-rats have very high affinity hemoglobin that can bind oxygen molecules in hypoxic environments that would be fatal to humans (Johansen et al. 1976).

### Intrinsic brain tolerance to hypoxia

**Fig. 2:**
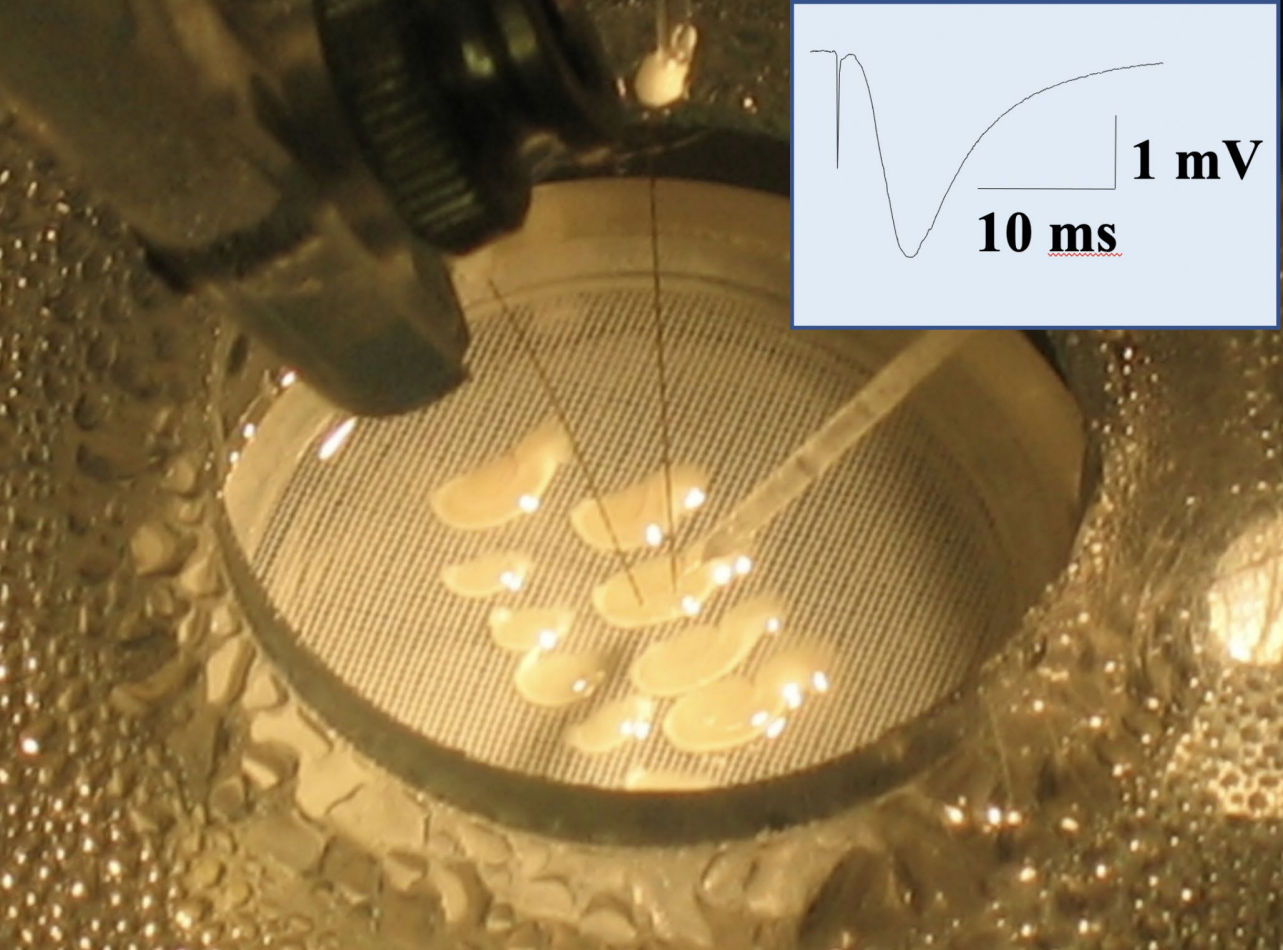
Hippocampal brain slices are pictured in a recording chamber where the concentration of O_2_ in the bath and atmosphere are precisely controlled. Stimulating and recording electrodes are positioned to generate and record evoked potentials (the summed activity of many neurons). The inset shows an example evoked potential. The initial, brief downward deflection is the stimulus artifact and indicates when the slice was stimulated. The large downward deflection is the evoked potential. Photo credit Thomas Park

Naked mole-rats demonstrate intrinsic brain tolerance to hypoxia. This was shown in experiments with hippocampal brain slices (Larson and Park 2009). Figure 2 shows hippocampal slices in the recording chamber. Note that 95% O_2_ is used in the bath and atmosphere of the chamber, which is the standard for brain slice experiments.

**Fig. 3:**
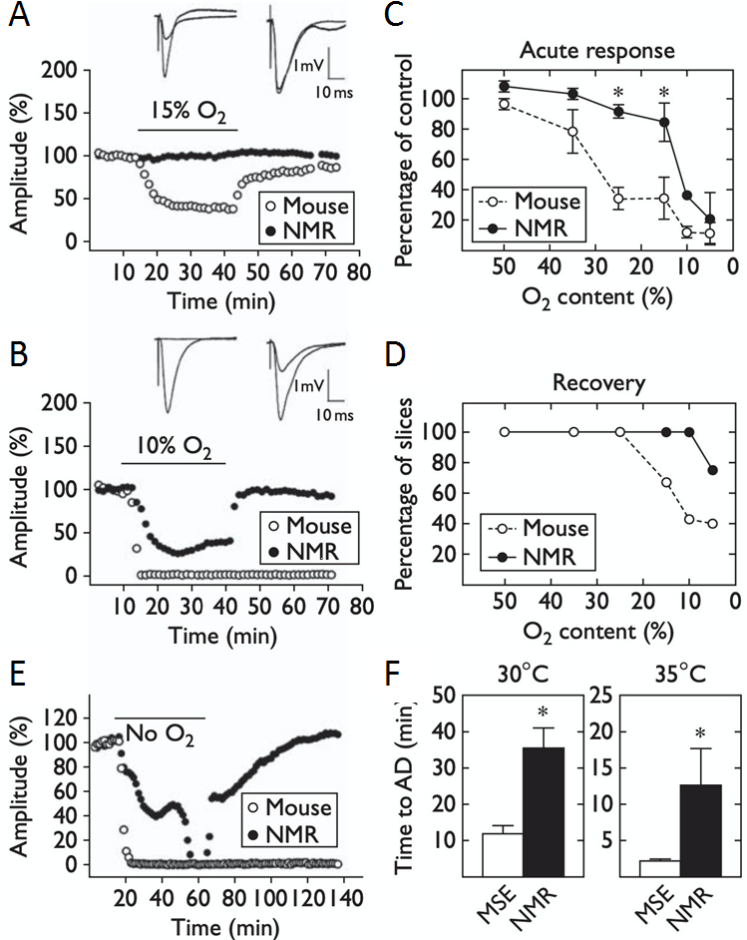
Intrinsic hypoxia tolerance in hippocampal brain slices from naked mole-rats compared to slices from mice. **A** and **B**, example curves from slices transiently exposed to 15% and 10% O_2_, respectively. **C** and **D**, summary data from naked mole-rat slices (n = 25) and mouse slices (n = 48). **E**, example curves from slices exposed to zero O_2_. **F**, time to complete loss of function during exposure to zero O_2_. Please see text for detail. This figure is from Larson and Park (2009)

Figure 3 shows the key results of that study. Figure 3 A shows the effects of exposing slices to 30 min of 15% O_2_ after achieving a stable baseline in 95% O_2_. The graph shows the amplitude of evoked field potentials triggered by a stimulating electrode. The stimulus and corresponding response took place every 20 s, and slices from mouse and naked mole-rat were tested side-by-side. The two example curves are from a mouse (open circles) and a naked mole-rat (NMR, closed circles) tested in the same bath solution. The horizontal bar labelled “15% O_2_” corresponds to the time when the O_2_ supply was changed from 95 to 15%. Exposure to 15% O_2_ caused the curve from the mouse to decline, indicating reduced synaptic function. Synaptic function recovered after 95% O_2_ was restored. The curve from the naked mole-rat was unaffected.

Figure 3B is in the same format as Fig. 3 A except that the period of hypoxia was more severe with only 10% O_2_. In this case, the slice from mouse completely lost function and did not recover. The slice from naked mole-rat declined in function but recovered after the hypoxic exposure. Figure 3 C shows the averaged decrease in evoked potential amplitude for various O_2_ concentrations, and Fig. 3D shows the percentage of slices that were able to recover from exposure to each O_2_ concentration. Clearly, the slices from naked mole-rat demonstrated significantly higher tolerance and higher recovery rate from hypoxia compared to slices from mouse.

Figure 3E shows the effects of exposure to zero O_2_ (anoxia). In this situation, functionality for the slice from naked mole-rat declined much slower than functionality for the mouse. Remarkably, the slice from naked mole-rat, but not mouse, recovered after O_2_ levels were restored. Figure 3 F shows the duration of 0% O_2_ exposure required to reach total loss of function (termed anoxic depolarization, AD) for mouse (MSE) and naked mole-rat (NMR). Data is shown for two bath temperatures. 35° C, which is close to mouse body temperature, and 30° C, which is close to naked mole-rat body temperature. In both cases, slices from naked mole-rats retained functionality significantly longer than slices from the mice.

### Upregulated expression of GluN2D, which is usually associated with hypoxia tolerance in neonates

**Fig. 4:**
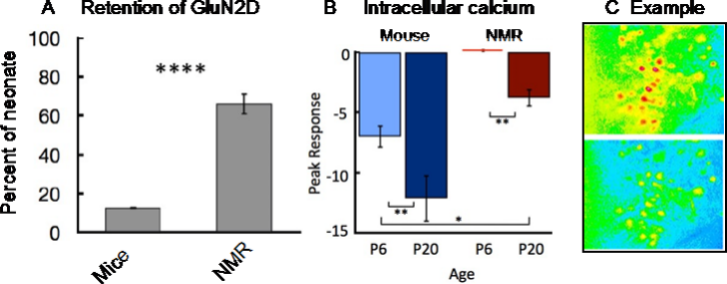
Retention of GLuN2D and reduced intracellular calcium from hypoxia in naked mole-rats. **A**. The bars show the percent of GLuN2D retained into adulthood for mice and naked mole-rats. This panel is from Peterson et al. (2012b). **B**. Florescent imaging results are shown for hippocampal cells from young (post-natal day 6, P6) and adult-like (post-natal day 20, P20) mice and naked mole-rat. Both ages of mice showed more intracellular calcium from hypoxia compared to naked mole-rats. Note that more negative values correspond to more calcium. **C**. Example data from one experiment. Panels B and C are from Peterson et al. (2012a)

One factor likely contributing to intrinsic hypoxia tolerance in naked mole-rat brain is the high expression level of GluN2D in adults of this species. Hypoxia and the related decline in energy trigger a cascade of cellular processes resulting in most NMDA-type calcium channels opening which allows toxic levels of calcium into the cells (Peterson et al. 2012b and references therein). Brain cells from neonates lessen this effect because they express a substantial number of NMDA channels containing the 2D subunit (GluN2D), which open much less during hypoxia (Bickler et al. 2003). In most mammals, the expression of GluN2D decreases precipitously postnatally (Laurie et al. 1997). Peterson et al. (2012b) found that adult naked mole-rats retain 66% of neonatal levels of GluN2D whereas adult mice only retain 13% of neonatal levels (Fig. 4 A).

NMDA-type calcium channels open when glutamate binds to them. Interestingly, Cheng et al. (2022) found that overall levels of glutamate decrease significantly in the brain of naked mole-rats exposed to hypoxia compared to mice. Decreasing glutamate levels is likely another way that naked mole-rats reduce calcium entry into cells to achieve tolerance to hypoxia.

### Reduced intracellular calcium accumulation from hypoxia

Subsequent experiments using calcium imaging showed that hippocampal cells from naked mole-rats displayed much less intracellular calcium from hypoxia compared to cells from mice (Fig. 4B,C). The bars in Fig. 4B indicate the average percent change in calcium-mediated fluorescence, with negative values corresponding to an increase in calcium (calcium decreases the fluorescent signal). An example from one experiment is shown in Fig. 4 C., which displays hippocampal cells before (top) and during (bottom) hypoxia.

### Whole animal tolerance to hypoxia and anoxia

**Fig. 5:**
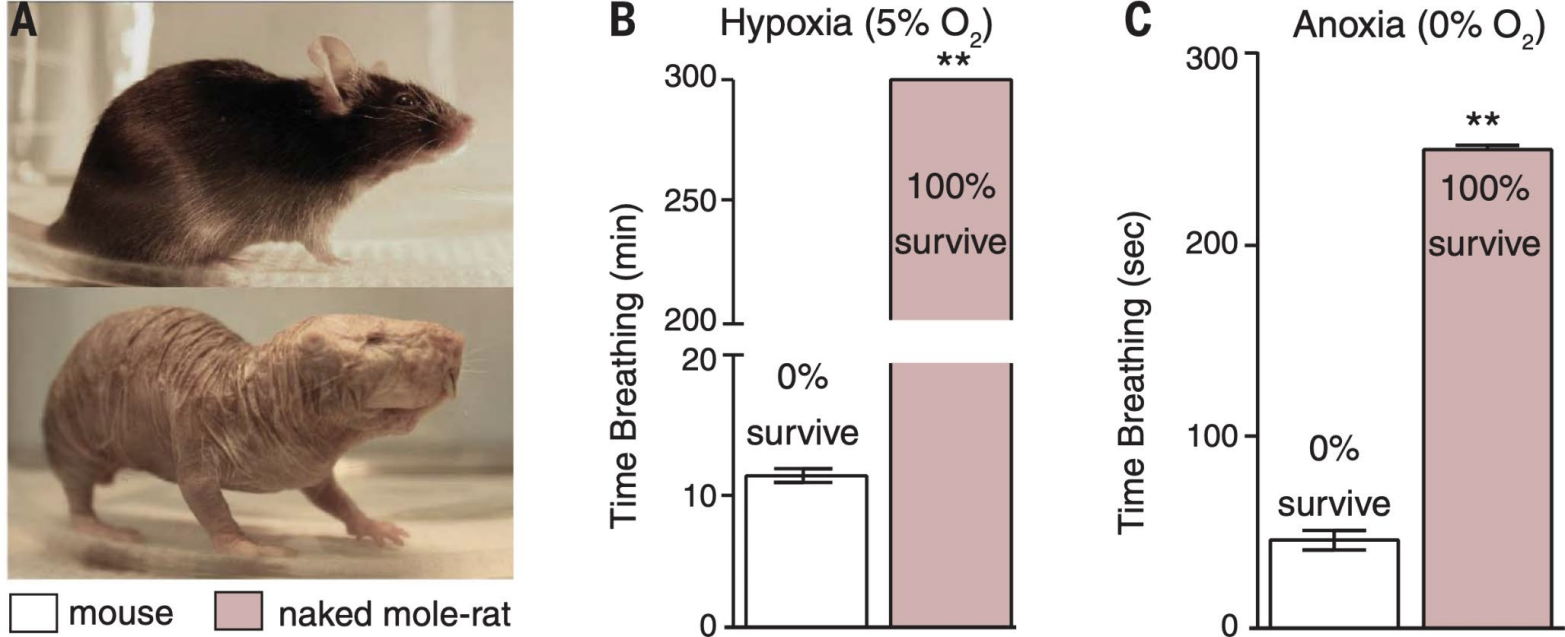
In vivo (whole animal) exposure to hypoxia (5% O_2_) and anoxia (0% O_2_) showed that naked mole-rats were extremely tolerant to these conditions compared to mice. Please see text for details. This figure is from Park et al. (2017)

The previous findings – reduced need for physiological thermoregulation, reduced resting metabolic rate, hemoglobin with a high affinity for O_2_, and intrinsic brain tolerance to hypoxia – set the stage for testing the whole animal. Testing whole animals revealed that naked mole-rats were many times more tolerant to both hypoxia and anoxia compared to mice (Park et al. 2017). Figure 5 shows the results of testing naked mole-rats and mice in atmosphere chambers where O_2_ concentrations were precisely controlled. Figure 5 A illustrates the similarity in size of a mouse and a naked mole-rat. Figure 5B shows the results of testing mice and naked mole-rats in hypoxia (5% O_2_). On average, the mice did not survive more than 12 min whereas the naked mole-rats were able to survive for 5 h. This experiment was arbitrarily stopped at the 5-hour point.

The results from testing animals in anoxia (0% O_2_) are shown in Fig. 5 C. During anoxia, both the mice and the naked mole-rats lost consciousness and ceased voluntary movements in about 30 s. Respiration attempts were monitored, and an animal was removed from the atmosphere chamber if there was no respiration attempt for 20 s for mice or 60 s for naked mole-rats. On average, the mice made their last respiration attempt after about 40 s into the exposure, whereas the naked mole-rats made their last respiration attempt after about 240 s (4 min). Remarkably, all of the naked mole-rats survived, even after an additional minute under anoxia, whereas none of the mice survived a much shorter exposure.

**Fig. 6:**
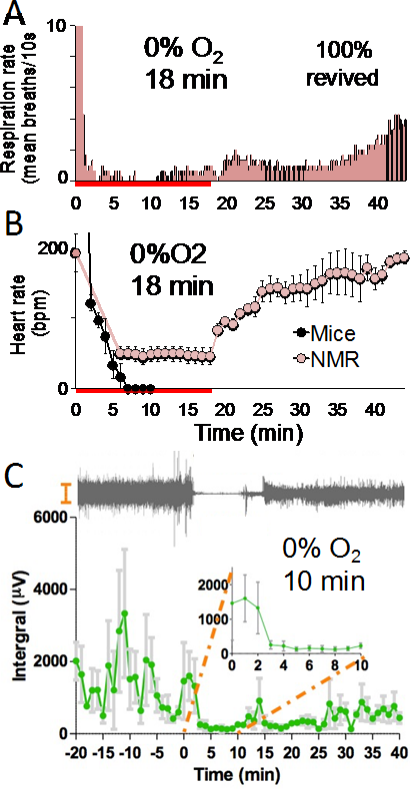
Physiological responses to anoxia. **A** and **B**. Average respiration rate (A) and average heart rate (B) dramatically decreased during 18 min of anoxia (N = 3). The red bar on the X-axis corresponds to when the animals were exposed to anoxia. These figures are from Park et al. (2017). **C**. EEG data shows that average brain activity also dramatically decreased during anoxia (N = 3). This figure is from Park et al. (2021)

The experiments described above showed that naked mole-rats could survive 5 min of exposure to anoxia (4 min to the last breath plus an additional minute before they were returned to room air (normoxia)). However, the next set of experiments revealed that 5 min was an underestimate of their capabilities. For the next experiments, respiration rate and heart rate were recorded, and naked mole-rats were exposed to anoxia for either 10, 18, or 30 min. Figure 6 A shows the results for respiration rate from the 18-minute exposure. The data is represented as average number of breaths per 10 s intervals. Respiration rate declined precipitously within the first few minutes of exposure, remained very low during the exposure, and then increased slowly when the atmosphere was returned to normoxia.

During these experiments, heart rates were simultaneously recorded from the same animals using an ECGenie recording system (Mouse Specifics, Inc.) where the animal’s feet were placed onto a platform with embedded electrode pads. Figure 6B shows average heart rate data from naked mole-rats and mice. For mice, the amplitude of the electrocardiogram trace declined into the noise after about 6 min in anoxia. In contrast, the average heart rate for the naked mole-rats declined from about 200 beats per minute in normoxia to about 50 beats per minute in anoxia. The average heart rate for naked mole-rats remained remarkably stable for the duration of the anoxia exposure, and then when the atmosphere was returned to normoxia, the heart rate gradually returned to baseline over the next 20 min. The end point of this experiment was when each naked mole-rat righted itself and walked off of the electrocardiogram contact pads.

Figure 6 C shows electroencephalography (EEG) data from a different cohort of naked mole-rats. Recordings were made in normoxia for 20 min prior to anoxia (-20 to 0 on the x-axis). In normoxia, brain activity was robust and quite variable. However, when the animals were exposed to anoxia, activity became extremely reduced (time 0 to 10 on the x-axis). The inset shows an expanded view of the time when they were in anoxia. Above the graph is an example EEG trace from one animal.

Cessation of voluntary movements and greatly reduced respiration rate, heart rate, and brain activity are reminiscent of a state called suspended animation (Blackstone et al. 2005; Blackstone and Roth, 2007), where biological functions are slowed to preserve physiological capabilities (Safar et al. 2000). This idea is consistent with the finding that naked mole-rats suppress their metabolism by up to 85% in acute severe hypoxia (Pamenter et al. 2018). The ability to go into a suspended animation state should be a great benefit to naked mole-rats for conserving energy during times of very low oxygen availability.

### Metabolic re-wiring

**Fig. 7:**
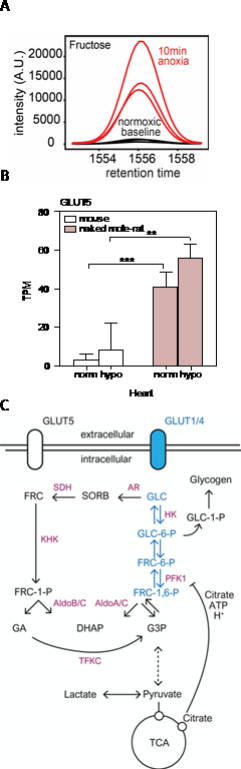
Switch to fructose metabolism under hypoxia. **A**. A chromatogram showing a fructose peak in the naked mole-rat under normoxic conditions (black) and its increase under anoxic conditions (red). This panel is from Park et al. (2017) (B) Level of Glut5 transcript is over 10-fold higher in the naked mole-rat heart compared to the mouse but its level of expression is unchanged under hypoxia. This panel is from Reznick et al. (2021). **(C)** Differences between fructose and glucose metabolism. Fructose is transported into the cell by the fructose-specific transporter GLUT5 whereas glucose enters the cell via other transporters including insulin-independent GLUT1 and insulin-dependent GLUT4. Fructose is phosphorylated by fructokinase (KHK) to generate fructose-1-phosphate (F1P), which consumes ATP and phosphate. F1P is then cleaved to glyceraldehyde (GA) and dihydroxyacetone phosphate (DHAP) by aldolase B or C (AldoB and C) after which the metabolism of glucose and fructose converge. Unlike glycolysis, fructolysis bypasses phosphofructokinase (PFK1), a rate-limiting step in glycolysis, to bypass the feedback inhibition at PFK1. PFK1 is a rate-limiting enzyme of glycolysis and its activity is blocked by high levels of ATP, citrate and low pH. KHK is much faster than hexokinase (HK) in phosphorylating its substrate thus leading to rapid ATP depletion and phosphate consumption. This relieves the blockade at PFK1 and increases glucose uptake and metabolism

In order to sustain even the most minimal heart and brain function (Fig. 6), tissues demand energy in the form of ATP, which is primarily generated via the glycolytic pathway or oxidative phosphorylation in the mitochondria. When oxygen is limited, ATP can be generated via anaerobic glycolysis, a process far less efficient but circumventing the need for oxygen. Metabolite analysis in various tissues and blood in the naked mole-rat revealed an unexpected finding. When exposed to an atmosphere with low or no oxygen the naked mole-rat activates the synthesis and utilization of fructose and sucrose (Fig. 7 A). Sucrose (a disaccharide composed of fructose and glucose) and fructose accumulated to high levels in the blood and tissues in the naked mole-rat exclusively under hypoxia. These metabolites were not detected in mouse tissue undergoing a similar hypoxic challenge. To support the idea that naked mole-rats switch to fructose metabolism, fructose-1-phosphate, a metabolite specific to fructose metabolism was also detected (Park et al. 2017). These findings suggested that in response to low oxygen, naked mole-rats are able to produce fructose in tissues such as liver and kidney, export this metabolite into circulation and deliver it to vital organs such as the brain and heart where it is used in glycolysis to generate ATP.

Fructose metabolism is largely limited to several tissues in most mammals, primarily the liver, kidney and intestine. This is due to restricted expression of the fructose transporter GLUT5 (Km = 6mM) and the less efficient transporter GLUT2 (Km = 11mM) (Hannou et al. 2018), as well as fructolytic enzymes fructokinase (KHK), aldolase reductase (ALDOB and C) and triokinase (TKFC) (Hannou et al. 2018). In contrast to mice and humans, naked mole-rats express high levels of Glut5 and Khka and Khkc mRNA across all tissues (Park et al. 2017) (Fig. 7B). Surprisingly, high expression of fructose-specific genes was observed in tissues from animals exposed to chronic normoxia suggesting that the naked mole-rat transcriptome is remodeled to enable efficient fructose uptake and utilization at all times and is not regulated by oxygen levels. Enhanced fructose metabolism under hypoxia seems therefore to be dependent on fructose availability rather than ability to transport and metabolize it. Indeed, increase in fructose and sucrose occurred only under low oxygen conditions. Under normoxic conditions, the levels of fructose and sucrose were very similar between mouse and naked mole-rat in all tissues tested.

Glucose is initially metabolized by the glycolytic pathway. Phosphofructokinase (PFK1) is the most critical regulatory enzyme in glycolysis, allosterically inhibited by ATP, citrate and lactate (Costa Leite et al. 2007; Kemp and Foe 1983). Under hypoxia, as lactate accumulates due to anaerobic glycolysis, PFK1 enzyme becomes inhibited leading to a slowing down of glycolytic flux and limiting the supply of ATP and glycolytic intermediates. Under these conditions, utilization of fructose as opposed to glucose can become beneficial to the cell. Fructolysis circumvents PFK1 inhibition by diverting metabolism towards the KHK enzyme which phosphorylates fructose to fructose-1-phosphate (F1P). F1P in turn is catalyzed to Dihydroxyacetone phosphate (DHAP) and Glyceraldehyde (GA) which enter glycolysis at a step downstream of the PFK1 enzyme (Fig. 7 C). The metabolism of fructose, unlike glucose is therefore an unchecked process, independent of the energy or metabolic state of the cell, enabling the cell to continue to attain ATP and glycolytic intermediates. A secondary advantage of fructose utilization has recently been demonstrated in APC^−/−^ tumor cells (Goncalves et al. 2019). Because the KHK enzyme has a very fast kinetic and is unregulated, the initial phosphorylation of fructose as it enters the cell depletes ATP dramatically. This manifests in a short relief of PFK1 inhibition, promoting glycolytic flux from glucose to generate sufficient ATP and glycolytic intermediates for cellular growth and maintenance (Van Den Berghe et al. 1977; Johnson et al. 2020; Mirtschink et al. 2018). The effect of fructose on glucose metabolism has not yet been investigated in the naked mole-rat however it is possible that similar to what was found in cancer, fructose may be relieving the block on glucose uptake and metabolism allowing for a greater glycolytic flux which would be advantageous under low oxygen conditions.

In a recent study, Hadj-Moussa et al. (2021) reported a downregulation in both Glut5 and Khk protein in the brain of naked mole-rats 4 h post-hypoxia at 7% oxygen. Furthermore, Khk was predicted to be a target of a differentially expressed miRNA (miRNA365) which was upregulated under hypoxia. Whether or not the downregulation of Glut5 and Khk protein observed in this study results in a reduction of fructose uptake or metabolism in the brain under hypoxia is yet undefined since neither of these parameters were examined in the study (Hadj-Moussa et al., 2021). Further studies need to be done to examine whether different grades of hypoxia elicit unique adaptive metabolic responses in the naked mole-rat.

## Extreme Hypercapnia Tolerance

Thus far, we have focused on hypoxia tolerance in the naked mole-rat, an example of extreme physiology related to evolving in a hypoxic atmosphere (at least in the nest chamber). The reason that the naked mole-rat burrow atmosphere is hypoxic is that many respiring animals in an unventilated burrow system deplete oxygen. The flip side of many respiring animals in the unventilated burrow system is accumulation of CO_2_. Breathing elevated concentrations of CO_2_ is problematic for several reasons including that it triggers tissue acidosis, pain, and pulmonary edema (Anton et al. 1992; Conlee et al. 2005; Lee and Pisarri 2001; Park et al. 2017; Russell et al. 1984). Naked mole-rats have evolved putative adaptations (A.K.A. extreme physiology) to deal with these challenges.

### Reduced behavioral aversion to CO_2_ and other painful fumes

**Fig. 8:**
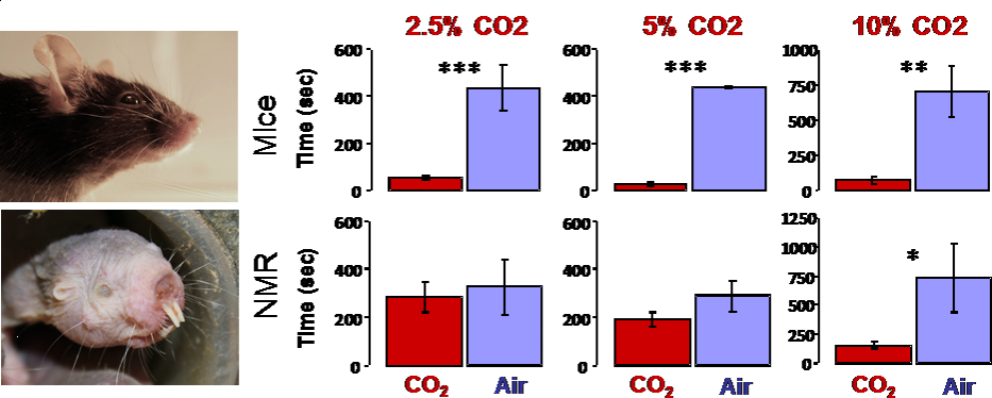
Behavioral avoidance to CO_2_. The bars correspond to time spent near a CO2 source versus time near a room air source. Mice (top panels) avoided all three concentrations of CO_2_ tested (2.5%, 5%, 10%), while naked mole-rats (bottom panels) only avoided the highest concentration (10%). This data is from Park et al. (2017)

The air that we breathe has a very low concentration of CO_2_, about 0.03%. In behavioral tests, Park et al. (2017) measured avoidance behavior to higher concentrations of CO_2_: 2.5%, 5%, and 10%. Mice and naked mole-rats were placed into a rectangular arena where they were free to move around. One end of the arena was infused with a given concentration of CO_2_ while the other end was infused at the same rate with room air. The researchers measured how much time each animal spent within 10 cm of both ends of the arena. The results showed that mice avoided all three concentrations of CO_2_, whereas the naked mole-rats only avoided the highest concentration, 10% CO_2_ (Fig. 8). This result is consistent with physiological data showing that naked mole-rats do not develop systemic acidosis from inhaled CO_2_ levels below 10% whereas mice develop substantial systemic acidosis at 1% (Park et al. 2017).

In addition to lack of aversion to CO_2_, a different study showed that CO_2_ concentrations up to 10% did not affect movement velocity, distance travelled, or exploration in either naked mole-rats or Damaraland mole-rats (Branigan et al. 2018).

**Fig. 9:**
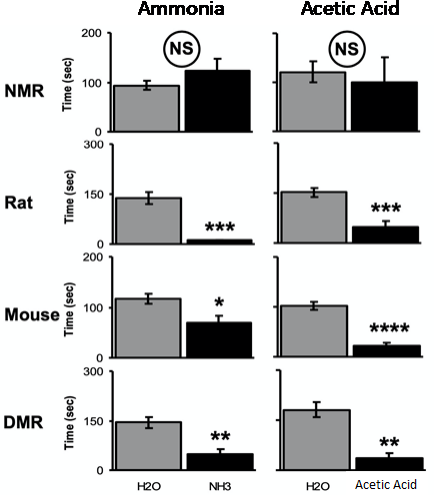
Behavioral avoidance to fumes from 10% ammonia and 10% acetic acid versus fumes from water. Data is from naked mole-rats and three comparison species, including another African mole-rat species, the Damaraland mole-rat (DMR). This figure is from LaVinka and Park (2012)

Also, LaVinka and Park (2012) showed that naked mole-rats did not avoid ammonia fumes or acetic acid fumes (Fig. 9). In addition to mice, this study also used laboratory rats and Damaraland mole-rats as comparison species. All three of the comparison species significantly avoided both ammonia and acetic acid fumes.

### Lack of CO_2_-induced pulmonary edema

**Fig. 10:**
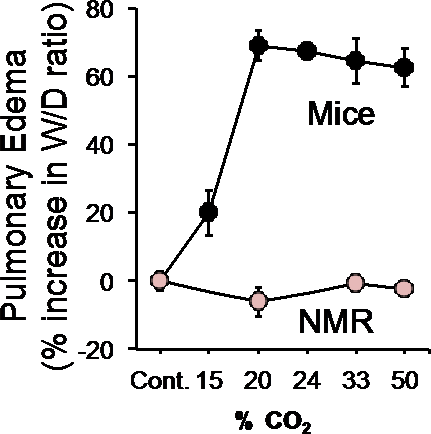
The graph shows pulmonary edema as a function of CO_2_ concentration for mice and naked mole-rats. The naked mole-rats show no pulmonary edema from breathing high concentration of CO_2_. This figure is from Park et al. (2017)

As mentioned earlier, CO_2_ not only causes pain, but it can also cause pulmonary edema. To determine if naked mole-rats were vulnerable to CO_2_-induced pulmonary edema, Park et al. (2017) placed animals into an environment chamber and exposed them to various concentrations of CO_2_ for 15 min. Then they quickly removed and weighed the lungs (wet weight), then dried the lungs and weighed them again (dry weight). This procedure generated a wet-to-dry weight ratio. They then calculated the percentage increase in the wet-to-dry ratio from CO_2_ exposure compared to control (room air) as a measure of pulmonary edema. In other words, how much excess fluid was in the lungs from breathing a given concentration of CO_2_. Figure 10 shows that mice are extremely vulnerable to CO_2_-induced pulmonary edema, while naked mole-rats showed no CO_2_-induced pulmonary edema, even at the highest concentration tested, 50% CO_2_.

### Lack of inflammatory and chemical pain

Hi levels of CO_2_ cause acidosis of the tissues, which in turn cause pain in the upper respiratory tract and pulmonary edema in the lungs. Both are mediated by a population of sensory nerves called C-fibers. C-fibers are unmyelinated pain fibers that not only transmit pain signals to the central nervous system (Smith and Lewin 2009), but they can release neuropeptides at the peripheral site of stimulation, which cause vasodilation and edema (Lee and Pisarri 2001). C-fibers are not restricted to the upper respiratory tract and lungs, rather they are found in many organs including the skin.

**Fig. 11:**
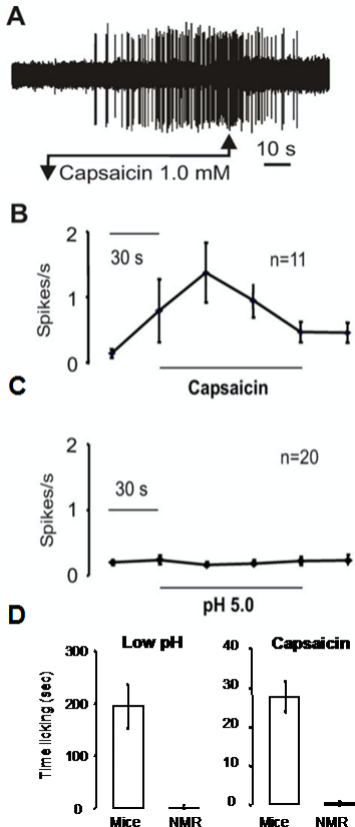
Naked mole-rat responses to acid and capsaicin. **A**. This is an example trace from a naked mole-rat C-fiber before, during, and after application of capsaicin solution. **B**. The graph shows averaged C-fiber responses to capsaicin. **C**. The graph shows averaged C-fiber responses to acid. **D**. The bar graphs show behavioral responses of mice and naked mole-rats to foot injection of acid and capsaicin injection

In the skin, C-fibers from naked mole-rats are completely insensitive to acid (Park et al. 2008; Smith et al. 2011). Figure 11 shows electrophysiological data from naked mole-rat C-fibers stimulated with acidic saline as well as another irritant, capsaicin, which is the spicy ingredient found in chili peppers. Figure 11 also shows behavioral responses to skin injections of acid and capsaicin solutions. Figure 11 A shows an example trace from a single C-fiber stimulated by capsaicin solution. Capsaicin elicited a barrage of action potentials, similar to what is seen in other species (Eigenbrod, 2019). Summary data from a population of C-fibers are shown in Fig. 11B, represented as action potentials (spikes) per second, showing a robust response to capsaicin. In contrast, population data from C-fibers tested with acidic saline (Fig. 11 C) show no physiological response.

The next question was, how would naked mole-rats respond behaviorally to application of capsaicin and acid in the skin? Fig. 11D shows behavioral results from mice and naked mole-rats. Animals received an injection of a small amount of irritant into the skin of one hind foot. Mice showed a robust licking response to both irritants whereas naked mole-rats showed virtually no response. It is noteworthy that naked mole-rats do respond to acute mechanical pain (pinch) and acute thermal pain (heat) the same as mice and other mammals (Park et al. 2008).

**Fig. 12:**
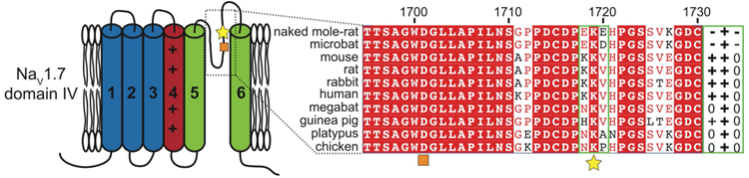
Sequence alignment of Na_v_1.7 for naked mole-rats and 9 other vertebrates. Note the EKE motif, yellow star has a negative charge in naked mole-rats (and microbats). This figure is from Smith et al. (2011)

Why are naked mole-rats physiologically and behaviorally insensitive to acid? Smith et al. (2011) found that two prominent acid receptors (acid-sensing ion channels and the transient receptor potential vanilloid-1 ion channel) are present and functional in C-fibers from naked mole-rats. However, that study went on to show that naked mole-rats have a mutation in their voltage gated sodium channel 1.7 (Na_v_1.7). In other mammals, Na_v_1.7 channels are located on the axons of pain fibers, and they are critical for propagating actions potentials initiated by acid. The mutation in naked mole-rat Na_v_1.7 causes a blockade of action potential when protons (acid) are present, but not other irritants such as capsaicin. Figure 12 shows a sequence alignment of Na_v_1.7 from naked mole-rats and nine other vertebrates. Compared to all of the other animals, except the microbat, the naked mole-rat sequence has a negatively charged EKE motif (star in Fig. 12). The charge of this motif is displayed to the right of the sequence alignment.

The mutation in naked mole-rat Na_v_1.7 can account for this species’ physiological and behavioral insensitivity to acid. However, it cannot account for the naked mole-rat’s behavioral insensitivity to capsaicin (and ammonia, Fig. 9). Recall that naked mole-rat C-fibers respond to capsaicin (Fig. 11 A,B), but intact animals do not react to capsaicin injection (Fig. 11D).

**Fig. 13:**
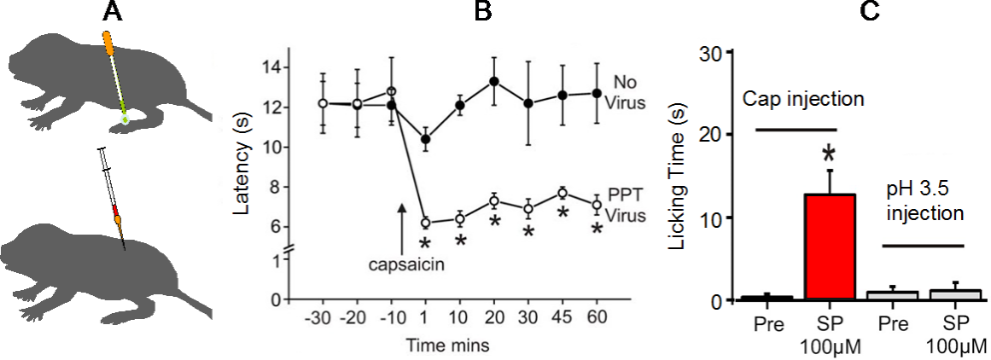
Introducing neuropeptides that naked mole-rats naturally lack can rescue pain behaviors. **A**. Neuropeptides were introduced via a transgenic herpes virus carrying the PPT gene (top) or via direct injection into the spinal cord (bottom). **B**. The PPT virus infected foot was enabled to be sensitized by topical application of capsaicin, as seen in other animals. **C**. After intrathecal injection of Substance P, naked mole-rats showed pain behaviors to foot injection of capsaicin. This data is from Park et al. (2008)

It turns out that naked mole-rat C-fibers lack neuropeptides (e.g. Substance P) (Park et al. 2003, 2008). The C-fibers of most other mammals release neuropeptides as well as the excitatory neurotransmitter glutamate at their synapses in the spinal cord (Smith and Lewin 2009). C-fibers from naked mole-rats only release glutamate. Park et al. (2008) showed that introducing one of the neuropeptides, Substance P, was sufficient to rescue pain behaviors from capsaicin (Fig. 13). Figure 13 A demonstrates the two methods of introducing Substance P. One method involved infecting one foot with a transgenic herpes virus carrying the preprotachykinin (PPT) gene for the neuropeptides that naked mole-rats naturally lack. The other method involved making intrathecal injections of Substance P into the spinal cord. Figure 13B shows latency for foot withdrawal to radiant heat one week after infecting one foot with the herpes virus. The first three data points show that the animals withdrew both the uninfected foot (labeled No Virus) and the infected foot (labeled PPT Virus) after about 12 s of heat application. However, when capsaicin was topically applied to the skin of the feet after the third data point, the infected foot showed robust sensitization from the capsaicin while the uninfected foot remained insensitive. Sensitization is indicated by the decrease in withdrawal latency. The sensitization by topical capsaicin of the infected foot is very similar to data from uninfected rats and mice (Yeomans et al. 1996). Figure 13 C shows the results for intrathecal injection of Substance P. Prior to the intrathecal injection, the naked mole-rats showed virtually no pain response (licking the injection site) from capsaicin injection into the skin of the foot (first bar, labeled Pre). However, after the intrathecal injection, the animals showed a clear pain response (second bar, labeled SP 100µM). Hence pain behavior from capsaicin was rescued by introducing Substance P into the spinal cord. A similar experiment using acid instead of capsaicin as the pain stimulus did not rescue pain behavior (bars labeled pH 3.5 injection) because the presence of Substance P would not change the way that the Na_v_1.7 channels are disabled by protons (acid).

**Fig. 14:**
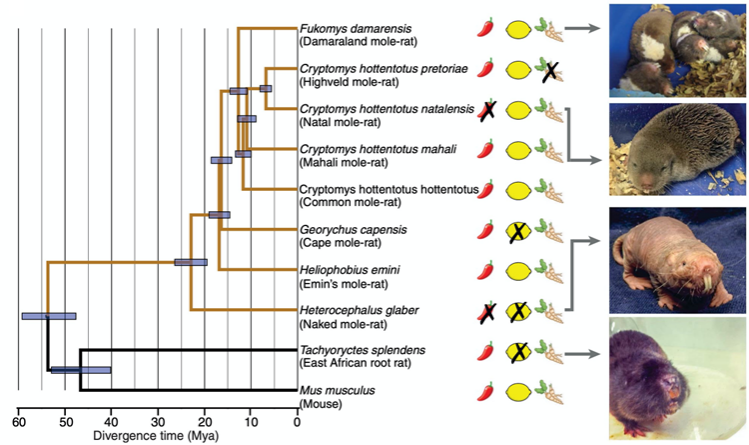
This figure shows a phylogenetic tree for the 10 African, subterranean rodent species tested for pain insensitivities by Eigenbrod et al. (2019). Two species showed insensitivity to capsaicin (X marks over the red chili pepper symbols). Three species showed insensitivity to acid (X marks over the yellow lemon symbols). One species showed insensitivity to AITC, (X marks over the wasabi root symbol). Mya = million years ago. This figure is from Eigenbrod et al. (2019)

Prior to 2019, the naked mole-rat was the only known mammal that was insensitive to capsaicin and the only known vertebrate that was insensitive to acid. However, a study by Eigenbrod et al. (2019) identified additional mammals with pain insensitivities. They tested ten African subterranean rodents (Fig. 14) and found two additional species that are insensitive to acid, one species that is insensitive to capsaicin, and one species that is insensitive to allyl isothiocyanate (AITC), the spicy ingredient in wasabi. The highveld mole-rat is the species that is insensitive to AITC. Interestingly, the highveld mole-rat shares its burrows with the Natal droptail ant, which has venom that activates the same receptors that respond to AITC, the TRPA1 receptor. Eigenbrod et al. (2019) showed that pain fibers in the highveld mole-rat overexpress the sodium leak channel NALCN, which dampens excitation from stimulating TRPA1 channels with AITC or ant venom, presumably an adaptation to living with stinging ants.

### Summary

Naked mole-rats show a robust tolerance to hypoxia, hypercapnia, and irritants including CO_2_, acid, capsaicin, and ammonia. Some mechanisms for these tolerances have been identified. Examples include modulating metabolic pathways to achieve tolerance to hypoxia, a mutation in a voltage gated sodium channel renders them insensitive to acid, and a lack of Substance P in peripheral nerves renders them insensitive to capsaicin. We believe that these adaptations reflect evolution in their extreme environment, akin to adaptations in other model organisms such as bats, barn owls, and weekly electric fish. We are confident that the naked mole-rat, a relatively new model organism, will continue to provide a valuable system for biologists, ecologists, and biomedical scientists.

## Electronic supplementary material

Below is the link to the electronic supplementary material.

Supplementary Material 1
