# Correction: Extreme Physiology Extreme Tolerance to Hypoxia, Hypercapnia, and Pain in the Naked Mole-Rat

**DOI:** 10.1007/s10974-023-09654-4

**Published:** 2023-09-11

**Authors:** Thomas J. Park, Jane Reznick

**Affiliations:** 1 Department of Biological Sciences and Laboratory of Integrative Neuroscience, University of Illinois at Chicago, Chicago, IL United States of America; 2 Cologne Excellence Cluster for Cellular Stress Responses in Aging-Associated Diseases (CECAD), Faculty of Medicine and University Hospital, University of Cologne, Cologne, Germany

**Correction to: Journal of Muscle Research and Cell Motility (2022) 44:61–72** **https://doi.org/10.1007/s10974-022-09623-3**

In original publication of the article Title was published incorrectly. The corrected Title is given in this correction. The original version has been corrected.

