# Supplementary material for: Extreme Physiology Extreme Tolerance to Hypoxia, Hypercapnia, and Pain in the Naked Mole-Rat
Source: J Muscle Res Cell Motil. 2022 Jul 19;44(2):61–72. doi: 10.1007/s10974-022-09623-3 (PMC10329625; doi:10.1007/s10974-022-09623-3)
Supplement: Supplementary file 1 — Supplementary Material 1 [file 10974_2022_9623_MOESM1_ESM.doc]

____________________________________________________________________________________

840 W. Taylor Street, (m/c 067)

Chicago, Illinois 60607-7060

JURE-D-22-00008

Re: Issue focused on Extreme Physiology	April 19, 2022

Dear Dr. Somlyo,

Accompanying this cover letter, please find our revised submission for the issue on extreme physiology titled “Extreme Tolerance to Hypoxia, Hypercapnia, and Pain in the Naked Mole-Rat.” Below is our point by point reply to the reviewers concerns.

Sincerely,

Thomas Park, PhD

Professor of Biological Sciences Associate Head of Department

**Reviewer #1:**

In this review the authors set out to summarize what is known regarding adaptations to life in hypoxia and hypercapnia in naked mole rats (NMRs). This is a fascinating study species with significant research potential, and I was excited to read this review. Although the manuscript is well-written, I was disappointed that the content unfortunately falls far short of providing a comprehensive review of the field. Instead, the authors essentially only discuss and cite their own work on these topics and ignore the large and growing body of work that has recently explored adaptations to these extreme environmental challenges in this species. There are also several instances where the authors make incorrect or sweeping statements that do not accurately capture our understanding of the physiology of this species. I encourage the authors to present a more balanced review of the state of the field or to change the title to indicate that they are only reviewing their own work.
*** We appreciate the feedback and we have made revisions throughout the text.**

Major Comments

-       Page 5 - Yes, naked mole-rats do behaviourally thermoregulate but the statement that they don't physiologically thermoregulate is not correct. They are not able to MAINTAIN their Tb very well when outside of their thermoneutral zone but they certainly spend a lot of energy trying to do so. Oiwa et al, Withers and Jarvis, Kirby et al, McNab, Cheng et al, and others have all demonstrated that NMRs maintain a body temperature well above ambient temperatures when held in temperatures below their thermoneutral zone. More importantly, several of these studies have measured metabolic rate and

demonstrated that NMRs have a higher metabolic rate in colder temperatures, which is fairly robust evidence that these animals use physiological thermoregulation. Please revise this paragraph to accurately present the current understanding of thermoregulation in this species.

*** We have revised this paragraph which now reads as follows: “Naked mole-rats employ at least two mechanisms to conserve oxygen-dependent energy. The first mechanism involves a reduced generation of body heat when they are within their thermoneutral zone (reviewed in Buffenstein et al., 2021a;b). Within their equatorial burrows, naked mole rats can find a desirable ambient temperature by moving to different depths of the burrow as the surface of the earth heats and cools over the course of the day and night (Jarvis, 1990). Holtze et al. (2017) found that burrow temperatures range over 24.6–48.8°C.**

**In other words, they behaviorally thermoregulate. Other mammals use physiological thermoregulation and expend a huge amount of energy to maintain their desired body temperature (Cannon and Nedergaard, 2011). Hence, by favoring behavioral thermoregulation, naked mole-rats greatly reduce their need for oxygen-dependent energy (Buffenstein and Yahav, 1991). Interestingly, in the laboratory, naked mole-rats exposed to cool temperatures (e.g. 20 degrees C) attempt to physiologically thermoregulate. However, they are unable to maintain their preferred temperature (Oiwa et al., 2020). Also interesting, within captive colonies maintained within their thermoneutral zone, naked mole-rats regulate their body temperatures within 1 degree C by huddling together (Yahav and Buffenstein, 1991). Another way that naked mole-rats behaviorally respond to hypoxia is that they progressively reduce activity in response to progressively more severe hypoxic atmospheres, presumably to save energy (Ilacqua et al., 2017).”**

-       Hemoglobin p50 and low basal metabolic rate are only a few of the physiological responses to hypoxia that have been described in this species. Consider a more complete summary of this area of adaptation. Behavioural responses to hypoxia might also be considered here.

*** The following is now included in the above paragraph: “Interestingly, in the laboratory, naked mole-rats exposed to cool temperatures (e.g. 20 degrees C) attempt to physiologically thermoregulate. However, they are unable to maintain their preferred temperature (Oiwa et al., 2020). Also interesting, within captive colonies maintained within their thermoneutral zone, naked mole-rats regulate their body temperatures within 1 degree C by huddling together (Yahav and Buffenstein, 1991). Another way that naked mole-rats behaviorally respond to hypoxia is that they progressively reduce activity in response to progressively more severe hypoxic atmospheres, presumably to save energy (Ilacqua et al., 2017).”**

-       Intrinsic brain tolerance: The authors spend 3 pages exhaustingly presenting and explaining the experimental preparation and results from Larson and Park 2009, which is a bit odd for a review. I suggest trimming this to a few sentences summarizing the key findings of this study. The space here would be better spent describing many of the

other interesting findings in NMR brain that support their intrinsic tolerance to hypoxia. Many studies have advanced this field in the past 12 years!

*** We reduced the text in this section as suggested.**

-       A similar comment applies to discussion of data in figure 6. It is important and interesting data but the authors should be able to summarize the key findings more succinctly and use the space to more thoroughly review what is known about adaptations to hypoxia in this species.
*** We reduced the text in this section as suggested.**

-       GluN2d expression: Glutamate levels in hypoxic NMR brain have been recently reported (Denu's group: Cheng et al, Metabolites 2022). As the trigger of NMDA receptor activity and a key step in hypoxic cell death in human brain, this is highly relevant to this section of the manuscript and should be included in this review.
*** We have added the following test and the reference to Cheng et al: “NMDA-type calcium channels open when glutamate binds to them. Interestingly, Cheng et al. (2022) found that overall levels of glutamate decrease significantly in the brain of naked mole-rats exposed to hypoxia compared to mice. Decreasing glutamate levels is likely another way that naked mole-rats reduce calcium entry into cells to achieve tolerance to hypoxia.”**

-       Page 15, fructose metabolism. Although the Science publication indicated that NMRs have a high endogenous expression of GLUT5 and KHK relative to mice, subsequent work from the Storey group (Hadj-Moussa et al 2021) demonstrated that both of these proteins are downregulated in brain of animals exposed to hypoxia in vivo. This finding should be presented and discussed given the context of this review. Please present a balanced evaluation of the available data.
*** We added the reference and the following text to this section: “In a recent study, Hadj-Moussa et. al. (2020) reported a downregulation in both Glut5 and Khk protein in the brain of naked mole-rats 4 hours post-hypoxia at 7% oxygen. Furthermore, Khk was predicted to be a target of a differentially expressed miRNA (miRNA365) which was upregulated under hypoxia. Whether or not the downregulation of Glut5 and Khk protein observed in this study results in a reduction of fructose uptake or metabolism in the brain under hypoxia is yet undefined since neither of these parameters were examined in the study (Hadj-Moussa et al., 2020). Further studies need to be done to examine whether different grades of hypoxia elicit unique adaptive metabolic responses in the naked mole-rat.”**

-       Aversion to CO2. Brannigan et al also explored behavioural impacts in high CO2 environments and should be discussed here. This study also explored DMRs and would fit in well with the LaVinka study discussed in the following paragraph.
*** We added the reference and the following text to this section: “In addition to lack of aversion to CO2, a different study showed that CO2 concentrations up to 10% did not affect movement velocity, distance travelled, or exploration in either naked mole-rats or Damaraland mole-rats (Branigan et al., 2018).”**

-       Acid pain. This is a fascinating area of study but the Park et al Science paper also showed that NMRs don't develop systemic acidosis at inhaled CO2 levels below 10%. Thus, regardless of cellular adaptations that limit their sensitivity to hypercarbia when/if it does occur in vivo, these animals must be mitigating the impact of inhaled CO2 to prevent acidification of the blood and tissues from occurring in the first place. This point should be discussed because the tissues are not likely experiencing hypercarbia at all in their natural burrows, as these are not likely to have CO2 levels as high as 10%!

*** We added the following text: “This result is consistent with physiological data showing that naked mole-rats do not develop systemic acidosis from inhaled CO_2_ levels below 10% whereas mice develop substantial systemic acidosis at 1% (Park et al., 2017).”**

-       The review ends very suddenly and without any concluding or summary statements. I recommend including a conclusions section to tie the whole thing together.
*** Thanks for the suggestion, we added a short summary at the end.**

Minor comments:

-       Intro (page 3). NMRs are not poikilothermic like reptiles and this is a misleading characterization. NMRs actively thermoregulate and use a significant amount of energy in this process (see Oiwa et al, 2020 and other refs above). Reptiles are not capable of generating heat. Please correct this sentence to refer to these animals appropriately as heterothermic.

*** We have revised the sentence, which now reads as follows: “In many ways they are heterothermic, taking on the temperature of their environment (Buffenstein et al., 2021a; Buffenstein et al., 2021b).”**

-       Page 5/6. Yes the air we breathe is 20% O2 near sea level but the brain doesn't experience O2 levels close to this normally. Brain O2 levels are likely 4-5% in vivo. I understand why it is necessary to perfuse brain slices with carbogen but I suggest changing this sentence to indicate the in vivo PO2 level of the tissue and not the inspiared O2 level in the air we breath, which is not really relevant to this statement or preparation.
*** We omitted the reference to 20% O2.**


**Reviewer #2:**

This is a well written summary and synthesis of a brilliant research program.  However, the authors undermine the evolutionary framework they wish to construct by ignoring much of the published data on naked mole-rats in their natural environment and citing secondary sources or out of date sources.   

Page 2 , 4, 19: The assumption that naked mole-rat burrows are hypoxic and hypercapnic is contradicted by field measurements.  Holtze et al 2017. 
*** We have added the following paragraph to page 4, including the reference to Holtze et al.: “It is important to note that Holtze et al. (2017) found that O2 and CO2 concentrations in naked mole-rat tunnels were very similar to those of the surface atmosphere. Those authors also suggested that hypoxic and hypercapnic conditions may well be a challenge in communal nest chambers where up to hundreds of individuals huddle together. Zions et al. (2020) showed that captive naked mole-rats spend more than 70% of their time in the nest chamber, which is substantial.”**

*** On page 19, we added: “(at least in the nest chamber).”**

Page 3:  The assertion that there is a soldier caste has been contradicted by Mooney et al 2015 and by Thorley et al 2018.

*** We added the following sentence and the reference to , which reviews the literature about casts including the Mooney et al 2015 and Thorley et al 2018 references: “It should be noted that there is some controversy over the division of labor between these casts (see Gilbert et al., 2020 for a review).”**

Page ¾:  The idea that small colonies of naked mole-rats would starve to death is contradicted by Braude, 2000 who reported on the frequency of small colonies in the wild.

*** Good point, we replaced “starve to death” with the following: “a small colony would be more challenged by burrowing through the soil to find food”**

Page 5:  Discussion of temperature also ignores more recent data from Holtze et al 2017.

*** We revised this paragraph to include the data from Holtze et al 2017, and we included the reference.**

Page 29:  Amazingly, naked mole-rat burrows are also frequently infested with ants!  See  Braude, Schilder, and Muli, 1999.

*** Thank you for this reference, we very much enjoyed reading it.**
